# Contrasting effects of phosphatidylinositol 4,5‐bisphosphate on cloned TMEM16A and TMEM16B channels

**DOI:** 10.1111/bph.13913

**Published:** 2017-08-10

**Authors:** Chau M Ta, Kathryn E Acheson, Nils J G Rorsman, Remco C Jongkind, Paolo Tammaro

**Affiliations:** ^1^ Department of Pharmacology University of Oxford Oxford UK; ^2^ OXION Wellcome Trust Initiative in Ion Channels and Disease University of Oxford Oxford UK

## Abstract

**Background and Purpose:**

Ca^2+^‐activated Cl^−^ channels (CaCCs) are gated open by a rise in intracellular Ca^2+^ concentration ([Ca^2+^]_i_), typically provoked by activation of G_q_‐protein coupled receptors (G_q_PCR). G_q_PCR activation initiates depletion of plasmalemmal phosphatidylinositol 4,5‐bisphosphate (PIP_2_). Here, we determined whether PIP_2_ acts as a signalling lipid for CaCCs coded by the TMEM16A and TMEM16B genes.

**Experimental Approach:**

Patch‐clamp electrophysiology, in conjunction with genetically encoded systems to control cellular PIP_2_ content, was used to define the mechanism of action of PIP_2_ on TMEM16A and TMEM16B channels.

**Key Results:**

A water‐soluble PIP_2_ analogue (diC8‐PIP_2_) activated TMEM16A channels by up to fivefold and inhibited TMEM16B by ~0.2‐fold. The effects of diC8‐PIP_2_ on TMEM16A currents were especially pronounced at low [Ca^2+^]_i_. In contrast, diC8‐PIP_2_ modulation of TMEM16B channels did not vary over a broad [Ca^2+^]_i_ range but was only detectable at highly depolarized membrane potentials. Modulation of TMEM16A and TMEM16B currents was due to changes in channel gating, while single channel conductance was unaltered. Co‐expression of TMEM16A or TMEM16B with a Danio rerio voltage‐sensitive phosphatase (DrVSP), which degrades PIP_2_, led to reduction and enhancement of TMEM16A and TMEM16B currents respectively. These effects were abolished by an inactivating mutation in DrVSP and antagonized by simultaneous co‐expression of a phosphatidylinositol‐4‐phosphate 5‐kinase that catalyses PIP_2_ formation.

**Conclusions and Implications:**

PIP_2_ acts as a modifier of TMEM16A and TMEM16B channel gating. Drugs interacting with PIP_2_ signalling may affect TMEM16A and TMEM16B channel gating and have potential uses in basic science and implications for therapy.

**Abbreviations**
CaCC: calcium‐activated chloride channel
DrVSP: Danio rerio voltage‐sensitive phosphatase
*E*_*rev*_: reversal potential
G_q_PCR: G_q_‐protein coupled receptors
IP_3_: inositol triphosphate
PIP_2_: phosphatidylinositol 4,5‐bisphosphate
PLC: phospholipase C
PIPK: PIP 5‐kinase type Iγ
*V*_*m*_: membrane potential

## Introduction

Calcium‐activated chloride channels (CaCCs) are anion channels that are gated open in response to an increase in intracellular free Ca^2+^ concentration ([Ca^2+^]_i_), and by changes in the cell membrane potential (*V*_*m*_) towards depolarized values (Hartzell *et al.,* 2005; Ferrera *et al.,* 2011; Huang *et al.,* 2012a). Thus, CaCCs provide a link between Ca^2+^ signalling and membrane electrical activity. CaCCs are present in a wide range of tissues and play diverse physiological roles including modulation of mucus secretion in epithelial cells, control of neuronal and cardiac excitability as well as modulation of smooth muscle contraction (Hartzell *et al.,* 2005; Ferrera *et al.,* 2011; Huang *et al.,* 2012a).

The *TMEM16* family encompasses genes coding for CaCCs, such as *TMEM16A* and *TMEM16B*, as well as a gene (*TMEM16F*) encoding a protein with reportedly combined ion channel and lipid scramblase activity (Pedemonte and Galietta, 2014; Picollo *et al.,* 2015). The TMEM16A and TMEM16B channels share significant (~55%) sequence homology and present similarities in their electrophysiological properties (Scudieri *et al.,* 2011). For example, TMEM16A and TMEM16B display very similar degrees of selectivity and permeability to a range of anions (Adomaviciene *et al.,* 2013). Furthermore, the TMEM16A and TMEM16B channels are activated within overlapping ranges of [Ca^2+^]_i_ (e.g. Adomaviciene *et al.,* 2013; Betto *et al.,* 2014; Cruz‐Rangel *et al.,* 2015). The TMEM16A and TMEM16B paralogues also share some pharmacological properties. For instance, both TMEM16A and TMEM16B are modulated in a complex manner by antracene‐9‐carboxilic acid (A9C) (Cherian *et al.,* 2015; Ta *et al.,* 2016). These channels are inhibited by A9C *via* an open channel block mechanism while also being allosterically activated by the compound (Cherian *et al.,* 2015; Ta *et al.,* 2016). TMEM16A and TMEM16B are also blocked by other commonly used Cl^−^ channel blockers such as 4,4′‐diisothiocyano‐2,2′‐stilbenedisulfonic acid (DIDS) and niflumic acid with comparable potencies (Bradley *et al.,* 2014; Liu *et al.,* 2015; Pifferi *et al.,* 2009). In contrast, a recently identified drug (Ani9) selectively inhibited TMEM16A, with no significant block of TMEM16B (Seo *et al.,* 2016).

TMEM16A and TMEM16B differ in terms of their expression profiles and physiological roles. TMEM16A is involved in functions such as transepithelial Cl^−^ transport (Kunzelmann *et al.,* 2012; Scudieri *et al.,* 2012; Huang *et al.,* 2012a) and in the modulation of smooth muscle tone (Davis *et al.,* 2010; Manoury *et al.,* 2010; Thomas‐Gatewood *et al.,* 2011; Heinze *et al.,* 2014; Wang *et al.,* 2015). Conversely, TMEM16B is chiefly involved in the control of sensory processes including olfaction and vision (Stephan *et al.,* 2009; Stohr *et al.,* 2009; Hengl *et al.,* 2010; Pietra *et al.,* 2016) and is expressed in neuronal and glial cells (Ayoglu *et al.,* 2016). In spite of participating in somewhat distinct physiological functions, TMEM16A and TMEM16B appear to be modulated by common signalling pathways. It is well established that activation of G_q_‐protein coupled receptors, such as α_1_‐adrenoceptors and P2Y receptors, leads to activation of phospholipase C (PLC) that breaks down phosphatidylinositol 4,5‐bisphosphate (PIP_2_) and leads to the formation of inositol triphosphate (IP_3_). TMEM16A and TMEM16B channels can be activated by an IP_3_‐mediated increase in [Ca^2+^]_i_ (Hartzell *et al.,* 2005; Ferrera *et al.,* 2011; Huang *et al.,* 2012a). Understanding whether agonist‐induced changes in PIP_2_ levels also participate in the control of the activity of TMEM16A and TMEM16B channels is an important question in the cellular physiology of CaCCs. Indeed, PIP_2_ is known to modulate the activity of a variety of ion channel types (Suh and Hille, 2008; Hille *et al.,* 2015).

A recent study presented biochemical evidence that PIP_2_ binds to both cloned and native smooth muscle TMEM16A channels (Pritchard *et al.,* 2014). This study also included functional evidence that PIP_2_ modulates native CaCC currents in rat isolated, pulmonary artery smooth muscle cells (rPASMCs). For instance, inclusion of diC8‐PIP_2_, a water‐soluble PIP_2_ analogue, into the pipette solution led to a decrease in whole‐cell CaCC current in rPASMCs. Thus, it was proposed that PIP_2_ has an inhibitory effect on the native CaCC current in rPASMCs. Whether PIP_2_ functionally modulates cloned TMEM16A channels remains to be established. Furthermore, the possible modulation of the closely related TMEM16B channels by PIP_2_ has never been tested. Studying PIP_2_ modulation of cloned TMEM16A and TMEM16B channels in a heterologous expression system allows the underlying molecular mechanism to be examined in the absence of additional tissue‐specific modulatory pathways.

Here, we showed that cloned TMEM16A and TMEM16B channels are differentially modulated by PIP_2_, being activated and inhibited by this lipid respectively. The effect of PIP_2_ on TMEM16A channels was especially pronounced in the low μM range of [Ca^2+^]_i_ and was observed at negative as well as positive *V*_*m*_. In contrast, the effects of PIP_2_ on TMEM16B did not differ significantly over a wide range of [Ca^2+^]_i_ but was only detectable at highly depolarized *V*_*m*_ (≥50 mV). Thus, PIP_2_ may modulate TMEM16A under resting conditions as well as during membrane depolarization. In contrast, TMEM16B may be modulated only at highly depolarized *V*_*m*_, which might be reached by some types of excitable cells during action potential firing, especially during pathological conditions associated with elevations of the action potential peak. Identification of these new regulatory mechanisms highlights novel pathways for potential pharmacological intervention; small molecules that affect PIP_2_ metabolism or directly interfere with PIP_2_ binding/transduction on TMEM16A or TMEM16B channels could affect channel gating and serve as novel channel modulators.

## Methods

### Cell culture and transfection

This study involved (i) mouse TMEM16A [isoform (ac) (Caputo *et al.,* 2008)] channels; (ii) mouse TMEM16B [isoform A (Ponissery Saidu *et al.,* 2013)] channels; (iii) PIP 5‐kinase type Iγ (PIPK) (provided by Y. Aikawa and T.F. Martin, University of Wisconsin, Madison, WI) each subcloned into the pcDNA3.1 vector; and (iv) Danio rerio voltage‐sensitive phosphatase (DrVSP) subcloned into pIRES‐EGFP vector (provided by Prof Y. Okamura, Osaka University). Site‐directed mutagenesis was performed as described previously (Tammaro and Ashcroft, 2009). HEK‐293T cells were cultured as previously described (Smith *et al.,* 2013) and transfected with 0.6 μg of TMEM16A or TMEM16B, 1 μg of DrVSP or PIPK and 0.2 μg of CD8 constructs using Fugene HD (Promega, UK) according to the manufacturer's instructions. Cells were used ~12–36 h after transfection. Transfected cells were visualized using the anti‐CD8 antibody‐coated beads method (Jurman *et al.,* 1994). Cells expressing CD8 were randomly selected for patch‐clamp recordings.

### Electrophysiology

TMEM16A and TMEM16B currents were measured with the whole‐cell or inside‐out configuration of the patch‐clamp technique as detailed in the Supplementary Information.

### Composition of solutions

The extracellular solution contained (mM): 150 NaCl, 1 CaCl_2_, 1 MgCl_2_, 10 glucose, 10 D‐mannitol and 10 HEPES; pH was adjusted to 7.4 with NaOH. The intracellular solution contained (mM): 130 CsCl, 10 EGTA, 1 MgCl_2_, 10 HEPES and 8 CaCl_2_ to obtain ~0.3 μM of [Ca^2+^]_i_; pH was adjusted to 7.3 with NaOH. Nominally, Ca^2+^‐free solution was obtained by omitting CaCl_2_. The intracellular solutions containing ~0.6, ~1, ~2 and ~78 μM [Ca^2+^]_i_ were obtained by replacing EGTA with equimolar H‐EDTA and by adding 2.1, 3.1, 4.8 and 9 mM CaCl_2_ respectively. In the experiments involving recovery of the DrVSP‐mediated modulation of TMEM16A and TMEM16B currents, MgATP (1 mM) was included in the pipette solution.

The water‐soluble PIP_2_ analogue diC8‐PIP_2_ (Echelon Biosciences, Salt Lake City, UT) was dissolved in an aqueous stock solution at 5 mg·mL^−1^, aliquoted and kept at −20°C. An appropriate amount of these stock aliquots was added to electrophysiological solutions on the day of the experiment. The resulting working solutions had concentrations of 1 μg·mL^−1^ (1.17 μM), 3 μg·mL^−1^ (3.51 μM), 10 μg·mL^−1^ (11.7 μM), 30 μg·mL^−1^ (35.1 μM) or 100 μg·mL^−1^ (117 μM).

### Main stimulation protocols

#### Current versus diC8‐PIP_2_ concentration ([diC8‐PIP_2_]) relationship

**Figure 1:**
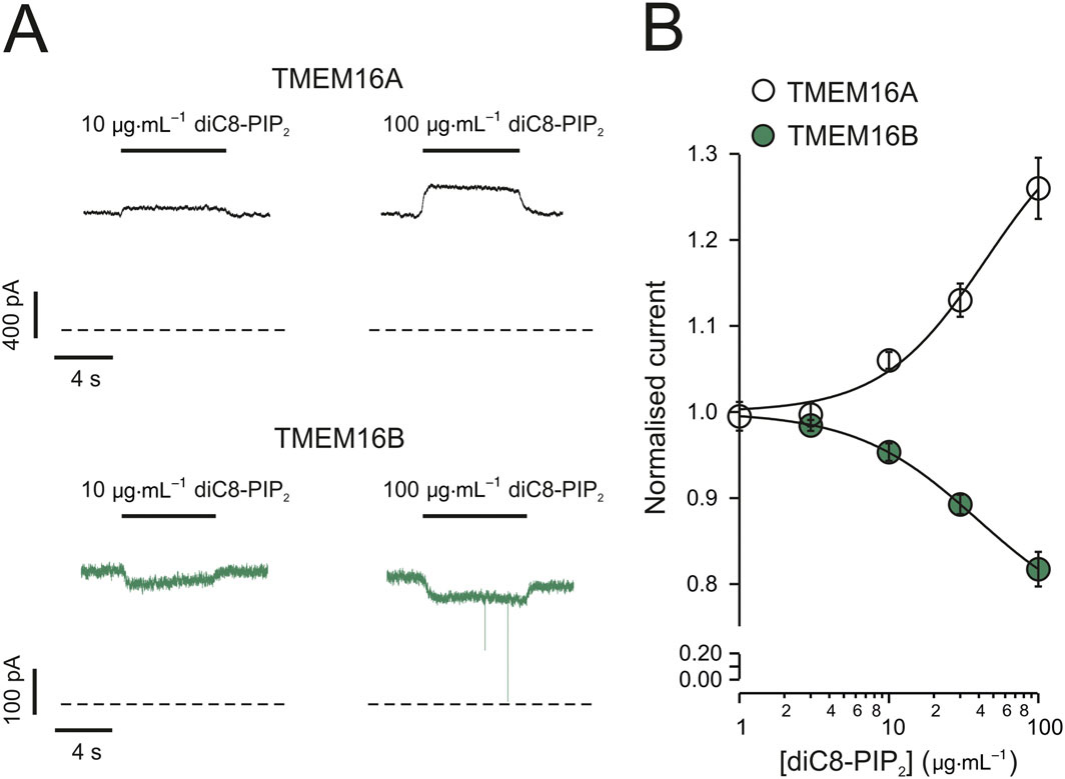
Effects of diC8‐PIP_2_ on TMEM16A and TMEM16B currents. (A) Currents recorded from inside‐out patches excised from HEK‐293T cells expressing either TMEM16A or TMEM16B, as indicated. diC8‐PIP_2_ was applied to the intracellular side of the patch, as indicated by the horizontal bars. The *V*_*m*_ was maintained at +70 mV for the entire duration of the recordings. [Ca^2+^]_i_ was 0.6 or 1 μM for experiments involving TMEM16A or TMEM16B respectively. Dashed lines represent zero‐current levels. (B) Mean relationships between diC8‐PIP_2_ concentration ([diC8‐PIP_2_]) and TMEM16A or TMEM16B currents, expressed relative to the current measured in the absence of diC8‐PIP_2_. The smooth curves through the points represent the best fits of the data using equation 1 (TMEM16A) or equation 2 (TMEM16B). The number of experiments was 12 (TMEM16A) or 9 (TMEM16B).

**Figure 2:**
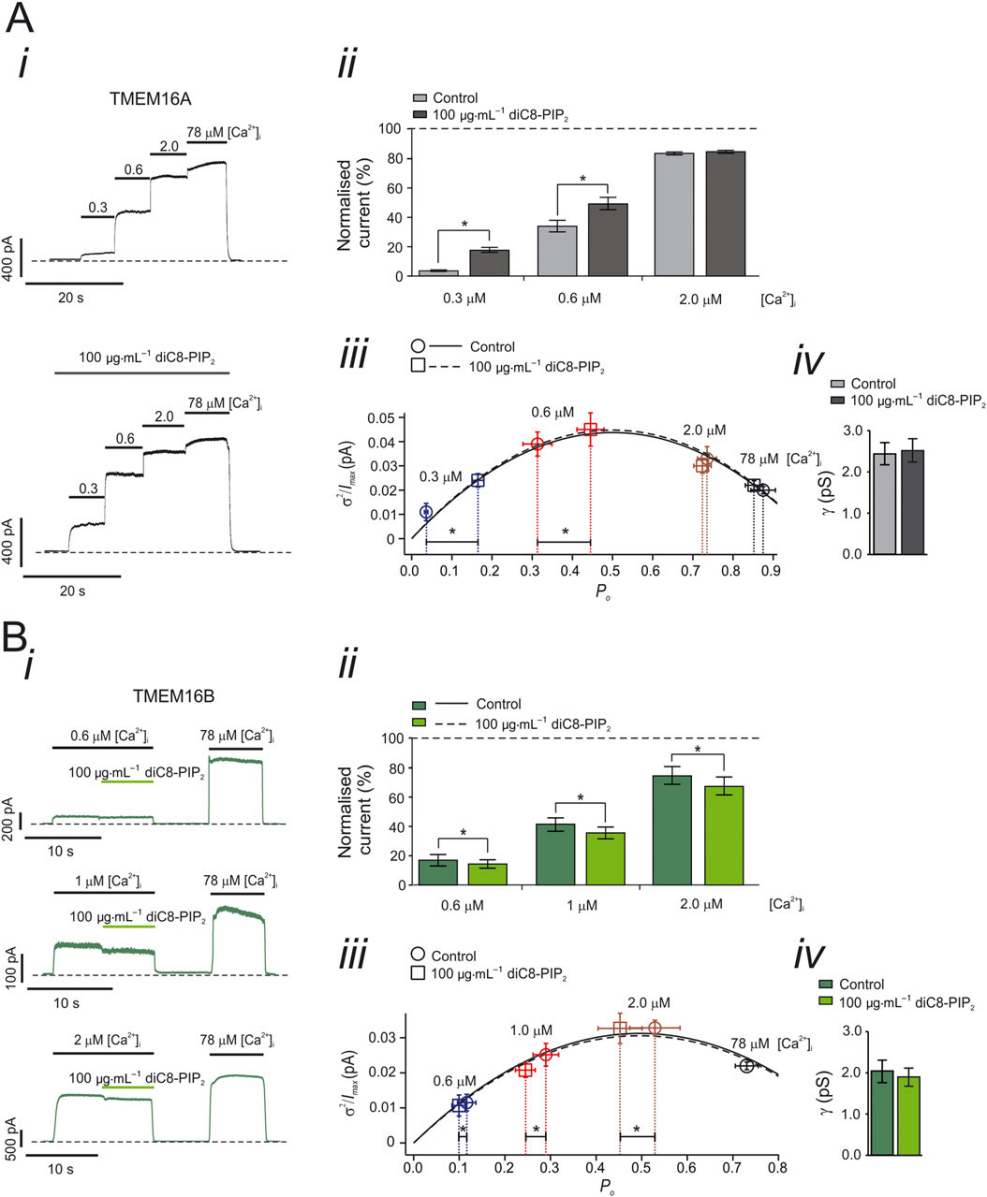
Effects of [Ca^2+^]_i_ on the sensitivity of TMEM16A and TMEM16B currents to intracellular diC8‐PIP_2_. (A, panel i) Currents recorded from an inside‐out patch excised from a HEK‐293T cell expressing TMEM16A in response to various [Ca^2+^]_i_, as indicated by the horizontal bars. diC8‐PIP_2_ [100 μg·mL^−1^ (117 μM)] was applied to the intracellular side of the patch, as indicated by the horizontal bar. The *V*_*m*_ was maintained at +70 mV for the entire duration of the recordings. Dashed lines represent zero‐current levels. (A, panel ii) Mean TMEM16A current amplitudes measured in the absence (control) or presence of diC8‐PIP_2_ and various [Ca^2+^]_i_. Currents measured at each [Ca^2+^]_i_ were normalized to the current measured in 78 μM [Ca^2+^]_i_. (A, panel iii) TMEM16A current variance (*σ* ^2^) normalized for the maximal current (*I*_*max*_) and plotted against the *P*_*o*_ for tracts of stationary currents recorded in the presence of various [Ca^2+^]_i_ and in the absence or presence of diC8‐PIP_2_. The parabolic lines are the best fit of the data using a quadratic function. (A, panel iv) Mean TMEM16A single channel conductance (*γ*) obtained from stationary noise analysis conducted in the presence or absence of diC8‐PIP_2_ [100 μg·mL^−1^ (117 μM)]. The number of experiments was 15 in each case. * *P* < 0.05 (paired *t*‐test). (B, panel i) Currents recorded from inside‐out patches excised from HEK‐293T cells expressing TMEM16B, in response to various [Ca^2+^]_i_ as indicated by the horizontal bars. diC8‐PIP_2_ [100 μg·mL^−1^ (117 μM)] was applied to the intracellular side of the patch as indicated by the horizontal bars. The *V*_*m*_ was maintained at +70 mV for the entire duration of the recordings. Dashed lines represent zero‐current levels. (B, panel ii) Mean TMEM16B current amplitudes measured in the absence (control) or presence of diC8‐PIP_2_ and various [Ca^2+^]_i_. Currents measured at each [Ca^2+^]_i_ were normalized to the current measured in 78 μM [Ca^2+^]_i_. (B, panel iii) TMEM16B current variance (*σ* ^2^) normalized for the maximal current (*I*_*max*_) and plotted against the *P*_*o*_ for tracts of stationary currents recorded in the presence of various [Ca^2+^]_i_ in the absence and presence of diC8‐PIP_2_. The parabolic lines are the best fit of the data using a quadratic function. (B, panel iv) Mean TMEM16B single channel conductance (*γ*) obtained from stationary noise analysis conducted in the presence or absence of diC8‐PIP_2_ [100 μg·mL^−1^ (117 μM)]. The number of experiments was 9 (experiments conducted in 0.6 μM [Ca^2+^]_i_), 12 (1.0 μM [Ca^2+^]_i_) or 21 (2.0 μM [Ca^2+^]_i_). * *P* < 0.05 (paired *t*‐test).

In our recordings conditions, HEK‐293T cells presented a small endogenous background current (Adomaviciene *et al.,* 2013; Ta *et al.,* 2016). To assess the sensitivity of TMEM16A and TMEM16B channels to diC8‐PIP_2_ during inside‐out patch‐clamp recordings (experiments of Figures 1 and 2), the currents were measured at +70 mV in nominally Ca^2+^‐free solution and in solutions containing a given [Ca^2+^]_i_. The small current measured in Ca^2+^‐free solution constitutes the endogenous background current and was subtracted offline from the current measured in the presence of Ca^2+^ before averaging the results. Thus, the resulting current represents the CaCC current component due to TMEM16A or TMEM16B channel activity.

TMEM16A and TMEM16B currents measured in the presence of diC8‐PIP_2_ (*I*_*diC8‐PIP2*_) were normalized to currents measured in the absence of diC8‐PIP_2_ (*I*_*0*_) and plotted against diC8‐PIP_2_ concentration ([*diC8‐PIP*_*2*_]). As outlined in the Results section, TMEM16A and TMEM16B currents were activated and inhibited by diC8‐PIP_2_ respectively.

The [*diC8‐PIP*_*2*_]‐response curves for TMEM16A were fitted with a Hill equation of the form

(1) $$\frac{I_{\mathrm{diC}8{\mathrm{PIP}}_{2}}}{I_{o}}=1+\frac{A_{\max}-1}{1+{\left(\frac{\left[\mathrm{diC}8\;-\;{\mathrm{PIP}}_{2}\right]}{K_{a}}\right)}^{h}},$$

where *A*_*max*_ is the maximal TMEM16A current activation, *K*_*a*_ is the [*diC8‐PIP*_*2*_] at which activation is half‐maximal and *h* is the Hill coefficient.

The [*diC8‐PIP*_*2*_]‐response curves for TMEM16B were fitted with a Hill equation of the form

(2) $$\frac{I_{{\mathrm{diC}8\mathrm{PIP}}_{2}}}{I_{o}}=\frac{1}{1+{\left(\frac{\left[\mathrm{diC}8\;-\;{\mathrm{PIP}}_{2}\right]}{K_{i}}\right)}^{j}},$$

where *K*_*i*_ is the [*diC8‐PIP*_*2*_] at which inhibition is half maximal and *j* is the Hill coefficient.

#### Current versus *V*_*m*_ relationship (I–V‐tail protocol)

Current versus *V*_*m*_ relationships were constructed by measuring currents in response to *V*_*m*_ steps of 1 s duration (test pulses) from −100 to +140 mV in 40 mV increments. Each test pulse was preceded by a *V*_*m*_ step to +70 mV of 1 s duration (pre‐pulse). Pulses were elicited every 2 s from a holding *V*_*m*_ of 0 mV. Steady‐state currents were measured at the end of the test pulses. For determination of the current reversal potential (*E*_*rev*_), instantaneous currents were estimated from extrapolation of single exponential fits of the test‐pulse currents to the beginning of each test pulse. These instantaneous current values were plotted as a function of the *V*_*m*_. The chord conductance and *E*_*rev*_ were determined from the linear fit of the instantaneous I–*V*_*m*_ relationship (Tammaro *et al.,* 2005; Adomaviciene *et al.,* 2013).

#### Stationary noise analysis

Stationary noise analysis (DeFelice, 1981) assumes that there are *N* independent and identical channels with a single conducting level, *i*. The macroscopic current (*I*) is given by

(3) $$I=\text{iN}P_{o}\;.$$

From binomial theory, the variance, *σ* ^2^, is related to *I* by

(4) $$\sigma^{2}=\mathrm{iI}+\frac{I^{2}}{N}\;.$$

Tracts (1–5 s duration) of stationary currents were measured at +70 mV and in different [Ca^2+^]_i_ in both the absence and presence of diC8‐PIP_2_. For each tract of current, the *σ* ^2^ and mean *I* were calculated. Background variance and current measured in 0 [Ca^2+^]_i_ were subtracted, and the *σ* ^2^ *‐I* plot was fit with equation 4 with *i* and *N* as free parameters. Single channel conductance (*γ*) was calculated by dividing *i* by the *V*_*m*_ at which the experiment was conducted_._ The *σ* ^2^ and *I* measured at each [Ca^2+^]_i_ were subsequently normalized for the estimated maximal current (*I*_*max*_, corresponding to *P*_*o*_ = 1) and averaged. In this way, the ordinate represents *σ* ^2^/*I*_*max*_ and the abscissa represents *P*_*o*_.

#### Recovery of TMEM16A currents from DrVSP‐mediated inhibition

A double‐pulse protocol was used to determine the time required for the response of TMEM16A or TMEM16B currents to recover following DrVSP activation during a 4 s pulse to +100 mV (conditioning pulse). The conditioning pulse was followed by a varying recovery period (3 to 55 s) at −50 mV and a subsequent 4 s test pulse to +100 mV. The effect of DrVSP was assessed by measuring the difference between the peak (*I*_*p*_) and the steady‐state (*I*_*ss*_) current elicited by each depolarizing pulse (*I*_*p*_ *− I*_*ss*_). The extent of recovery was expressed as the ratio of *I*_*p*_ *− I*_*ss*_ measured during a test pulse relative to that measured during the conditioning pulse. Time constant of recovery (*τ*_*r*_) was obtained by fitting a single exponential function to the relationship between extent of recovery and the duration of the recovery period.

### Data analysis

Data and statistical analysis comply with the recommendations on experimental design and analysis in pharmacology (Curtis *et al.,* 2015). Electrophysiological data were analysed with routines developed in the IgorPro (Wavemetrics, OR, USA) environment. Methods of analysis were established during study design, and prior to execution of the experiments, to remove possible operator bias. Statistical significance was determined with two‐tailed paired or unpaired *t*‐tests or one‐way ANOVA with Bonferroni's post test, as appropriate. For all statistical tests, *P*‐values < 0.05 were considered significant. Data are given as mean ± SEM alongside the number of experiments (*n*). The SPSS (version 22; SPSS Inc., Chicago, IL, USA) or Excel (Microsoft, USA) programmes were used for statistical analysis.

### Nomenclature of targets and ligands

Key protein targets and ligands in this article are hyperlinked to corresponding entries in http://www.guidetopharmacology.org, the common portal for data from the IUPHAR/BPS Guide to PHARMACOLOGY (Southan *et al.,* 2016), and are permanently archived in the Concise Guide to PHARMACOLOGY 2015/16 (Alexander *et al.,* 2015).

## Results

### Sensitivity of TMEM16A and TMEM16B channels to diC8‐PIP_2_

The project began by testing the sensitivity of cloned TMEM16A channels to diC8‐PIP_2_, a water soluble PIP_2_ analogue frequently used to investigate the sensitivity of ion channels to PIP_2_ (Suh and Hille, 2008; Hille *et al.,* 2015). TMEM16A currents were recorded in inside‐out patches excised from transfected HEK‐293T cells (Figure 1). In these experiments, *V*_*m*_ was kept constant at +70 mV and [Ca^2+^]_i_ was 0.6 μM. This [Ca^2+^]_i_ caused near half‐maximal TMEM16A channel activation (see below, Figure 2A). When diC8‐PIP_2_ was applied to the intracellular side of the patch, the TMEM16A current increased in a dose‐dependent manner up to a factor of 1.26 ± 0.04 (*n* = 12) in 100 μg·mL^−1^ (117 μM) diC8‐PIP_2_ (Figure 1). As described in greater detail below, the activating effect of PIP_2_ on TMEM16A channels became much more pronounced in the presence of lower [Ca^2+^]_i_. The TMEM16A sequence shares significant degree of homology with that of TMEM16B. We therefore tested the possibility that TMEM16B is also modulated by diC8‐PIP_2_. Because TMEM16B channels are less sensitive to activation by [Ca^2+^]_i_ than TMEM16A channels (Adomaviciene *et al.,* 2013; Scudieri *et al.,* 2013), the [Ca^2+^]_i_ in these experiments was elevated to 1 μM, a value causing approximately half‐maximal activation in our experimental conditions (Figure 2B). In this way, the effect of diC8‐PIP_2_ could be compared under conditions that cause similar extent of activation of TMEM16A and TMEM16B currents. Surprisingly, it was found that diC8‐PIP_2_ 100 μg·mL^−1^ (117 μM) inhibited TMEM16B currents by up to a factor of 0.82 ± 0.02 (*n* = 9) (Figure 1). The Hill fit of the relationships between the TMEM16A or TMEM16B currents and [diC8‐PIP_2_] yielded a *K*_*a*_ of ~45 μg·mL^−1^ (~53 μM) and *h* of ~1.2 (TMEM16A) (Table 1) and *K*_*i*_ of ~39 μg·mL^−1^ (~46 μM) and *j* of ~1.1 (TMEM16B) (Table 2).

**Table 1:** Parameters obtained from the Hill fit of the relationship between the extent of TMEM16A current activation and [diC8‐PIP_2_]

	*K*_*a*_ (μg·mL^−1^)	*K*_*a*_ (μM)	*h*	*A*_*max*_
TMEM16A	45 ± 8 (*n* = 12)	53 ± 9 (*n* = 12)	1.2 ± 0.4 (*n* = 12)	1.4 ± 0.1 (*n* = 12)

*A*_*max*_, maximal extent of current activation; *h*, Hill coefficient; *K*_*a*_, diC8‐PIP_2_ concentration producing half‐maximal activation of the channel (expressed in either in μg·mL^−1^ or in μM).

**Table 2:** Parameters obtained from the Hill fit of the relationship between the extent of TMEM16B current inhibition and [diC8‐PIP_2_]

	*K*_*i*_ (μg·mL^−1^)	*K*_*i*_ (μM)	*j*
TMEM16B	39 ± 2 (*n* = 9)	46 ± 2 (*n* = 9)	1.1 ± 0.1 (*n* = 9)

*j*, Hill coefficient; *K*_*i*_, diC8‐PIP_2_ concentration producing half‐maximal inhibition of the channel (expressed in either in μg·mL^−1^ or in μM).

### Effects of intracellular Ca^2+^ on the sensitivity of TMEM16A and TMEM16B channels to diC8‐PIP_2_

Intracellular Ca^2+^ levels are dynamically regulated in both excitable and non‐excitable cells. We asked if the effects of diC8‐PIP_2_ on cloned TMEM16A and TMEM16B channels varied depending on [Ca^2+^]_i_. TMEM16A currents were recorded from inside‐out patches exposed to different [Ca^2+^]_i_ in the absence or presence of 100 μg·mL^−1^ (117 μM) diC8‐PIP_2_ (Figure 2A*i*). Currents were normalized for the currents observed in the presence of 78 μM [Ca^2+^]_i_ as this Ca^2+^ level maximally activates TMEM16A channels (e.g. Adomaviciene *et al.,* 2013; Scudieri *et al.,* 2013). TMEM16A currents were not affected by 100 μg·mL^−1^ (117 μM) PIP_2_ under these conditions (Suppl. Figure S1). In the presence of 0.3 μM [Ca^2+^]_i_, diC8‐PIP_2_ increased the currents by a factor of 5.27 ± 1.29 (*n* = 15). In contrast, in the presence of 0.6 μM [Ca^2+^]_i_, diC8‐PIP_2_ caused an increase of the currents by a factor of 1.44 ± 0.17 (*n* = 15) while in 2 μM [Ca^2+^]_i_, there was no significant current activation (Figure 2A*ii*). Thus, the effects of diC8‐PIP_2_ on TMEM16A currents are strongly [Ca^2+^]_i_‐dependent.

To quantify the effects of [Ca^2+^]_i_ on the sensitivity of TMEM16B channels to diC8‐PIP_2_, a protocol distinct from the one adopted for TMEM16A channels was used. This was because we found that TMEM16B currents ran‐down more rapidly than TMEM16A currents when exposed to high [Ca^2+^]_i_. Thus, an experimental protocol of overall shorter duration was used to examine diC8‐PIP_2_ effect on TMEM16B currents. For each individual patch, the effect of diC8‐PIP_2_ was tested for an individual [Ca^2+^]_i_ and currents were normalized for the currents obtained in 78 μM [Ca^2+^]_i_ (Figure 2B*i*). This approach was justified by the fact that diC8‐PIP_2_ did not modulate TMEM16B currents measured in 78 μM [Ca^2+^]_i_ (Suppl. Figure S1). diC8‐PIP_2_ inhibited the currents by a factor of 0.85 ± 0.02 (*n* = 9) in 0.6 μM [Ca^2+^]_i_, 0.87 ± 0.02 (*n* = 12) in 1.0 μM [Ca^2+^]_i_, and 0.89 ± 0.01 (*n* = 21) in 2.0 μM [Ca^2+^]_i_ (Figure 2B*ii*). These degrees of inhibition were not statistically different from each other (one‐way ANOVA). Thus, the effects of diC8‐PIP_2_ on TMEM16B currents did not vary within the 0.6–2 μM [Ca^2+^]_i_ range, although there was no detectable diC8‐PIP_2_ inhibition in the presence of very high [Ca^2+^]_i_ (~78 μM), which resulted in maximal channel activation.

Changes in macroscopic current amplitude may be caused by changes in *i*, *P*_*o*_ or *N*. Changes in *N* are unlikely to occur in our experimental conditions (inside‐out patch‐clamp) as channel trafficking requires intracellular components that are presumably disrupted during patch excision. Stationary noise analysis revealed that application of diC8‐PIP_2_ [100 μg·mL^−1^ (117 μM)] resulted in an increase in *P*_*o*_ of TMEM16A channels. This increase was Ca^2+^ dependent: *P*_*o*_ was increased by 5.90 ± 1.20 (*n* = 15) fold in the presence of 0.3 μM [Ca^2+^]_i_ while in the presence of 0.6 μM [Ca^2+^]_i_ it increased only by a factor of 1.63 ± 0.17 (*n* = 15) and there was no detectable change in ≥2 μM [Ca^2+^]_i_ (Figure 2A*iii*). In contrast, *γ* of the TMEM16A channel was not affected by diC8‐PIP_2_ being ~2.5 pS in both the absence and presence of the lipid (Figure 2A*iv*). Stationary noise analysis also revealed that diC8‐PIP_2_ caused reduction in *P*_*o*_ of TMEM16B channels of a factor 0.87 ± 0.02 (*n* = 9), 0.85 ± 0.02 (*n* = 12) and 0.85 ± 0.02 (*n* = 21) in 0.6 μM, 1.0 μM and 2.0 μM [Ca^2+^]_i_, respectively (Figure 2B*iii*). The *γ* of the TMEM16B channel was ~2 pS in both the absence and presence of diC8‐PIP_2_ (Figure 2B*iv*). Thus, the changes in TMEM16A and TMEM16B current amplitudes caused by diC8‐PIP_2_ were due to changes in channel gating while *γ* was not affected.

### Effects of V_m_ on the sensitivity of TMEM16A and TMEM16B channels to diC8‐PIP_2_

**Figure 3:**
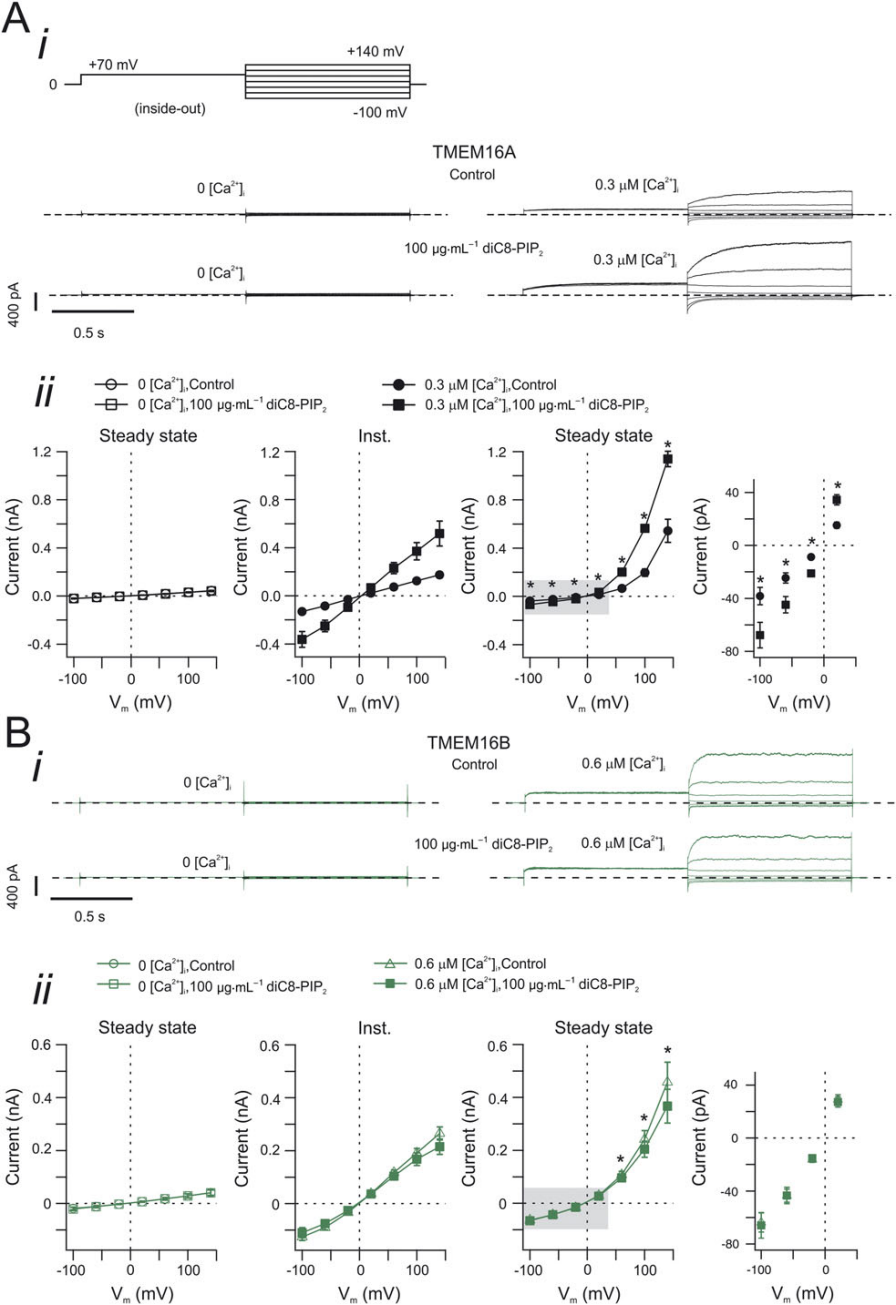
Effects of *V*_*m*_ on the sensitivity of TMEM16A and TMEM16B currents to intracellular diC8‐PIP_2_. (A, panel i) Currents recorded from inside‐out patches excised from HEK‐293T cells expressing TMEM16A. The stimulation protocol is shown in the top left corner. diC8‐PIP_2_ [100 μg·mL^−1^ (117 μM)] was applied to the intracellular side of the patch, as indicated. Dashed horizontal lines represent zero‐current level. [Ca^2+^]_i_ was 0 or 0.3 μM, as indicated. (A, panel ii) Mean instantaneous and steady‐state TMEM16A current versus *V*_*m*_ relationships measured in the absence (control) or presence of diC8‐PIP_2_ [100 μg·mL^−1^ (117 μM)], as indicated. The rightmost panel represents an expansion of the area highlighted in grey in the steady‐state current versus *V*_*m*_ relationship panel. The number of experiments was 6 (experiments conducted in 0 [Ca^2+^]_i_) or 9 (0.3 μM [Ca^2+^]_i_). (B, panel i) Currents recorded from inside‐out patches excised from HEK‐293T cells expressing TMEM16B. The stimulation protocol is shown in the top left corner in A, panel i. diC8‐PIP_2_ [100 μg·mL^−1^ (117 μM)] was applied to the intracellular side of the patch, as indicated. Dashed horizontal lines represent zero‐current level. [Ca^2+^]_i_ was 0 or 0.6 μM, as indicated. (B, panel ii) Mean instantaneous and steady‐state TMEM16B current versus *V*_*m*_ relationships measured in the absence (control) or presence of diC8‐PIP_2_ [100 μg·mL^−1^ (117 μM)], as indicated. The rightmost panel represents an expansion of the area highlighted in grey in the steady‐state current versus *V*_*m*_ relationships panel. The number of experiments was 5 (experiments conducted in 0 [Ca^2+^]_i_) or 15 (0.6 μM [Ca^2+^]_i_). * *P* < 0.05 (paired *t*‐test).

We next tested the effects of diC8‐PIP_2_ at various *V*_*m*_. During inside‐out patch‐clamp, a pre‐pulse of +70 mV was used to open TMEM16A or TMEM16B channels followed by a series of test pulses (I^_^V tail protocol (Figure 3A*i*)). TMEM16A and TMEM16B currents were recorded in the presence of 0.3 μM and 0.6 μM [Ca^2+^]_i_, respectively (Figure 3A*i* and Figure 3B*i*). These [Ca^2+^]_i_ were chosen as the effect of diC8‐PIP_2_ on TMEM16A is especially pronounced at 0.3 μM [Ca^2+^]_i_ while 0.6 μM [Ca^2+^]_i_ is a concentration that causes comparable basal activation of TMEM16B channels in our experimental conditions. The first observation was that the intracellular diC8‐PIP_2_ did not alter the *E*_*rev*_ of TMEM16A or TMEM16B current. In the absence and presence of 100 μg·mL^−1^ (117 μM) diC8‐PIP_2_ in the intracellular solution, the *E*_*rev*_ of TMEM16A current was 3.1 ± 1.1 mV (*n* = 9) and 3.4 ± 0.4 mV (*n* = 9), respectively (Figure 3A*ii*), and the *E*_*rev*_ of TMEM16B was −1.3 ± 1.0 mV (*n* = 15) and −1.2 ± 1.4 mV (*n* = 15), respectively (Figure 3B*ii*). These values are very close to the expected *E*_*rev*_ for Cl^−^ in our recording conditions (~1 mV). Thus, diC8‐PIP_2_ did not alter the TMEM16A and TMEM16B channel selectivity to ions. The slope of the instantaneous current *versus* voltage relationship provides a measure of the conductance of the membrane. In the absence and presence of 100 μg·mL^−1^ (117 μM) diC8‐PIP_2_ in the intracellular solution, the membrane conductance for patches expressing TMEM16A was significantly increased from 1.3 ± 0.2 nS (*n* = 9) to 4.1 ± 0.8 nS (*n* = 9) (*P* < 0.05, paired *t*‐test) while for patches expressing TMEM16B it was significantly decreased from 1.9 ± 0.2 nS (*n* = 15) to 1.6 ± 0.2 nS (*n* = 15) (*P* < 0.05, paired *t*‐test).

It is noteworthy that the diC8‐PIP_2_ promoted an increase in TMEM16A steady‐state current at all *V*_*m*_ (Figure 3A*ii)*. In contrast, the inhibitory effect of diC8‐PIP_2_ on TMEM16B steady‐state current was only observed at *V*_*m*_ > 50 mV (Figure 3B*ii*).

We finally examined the requirement for intracellular Ca^2+^ in the development of the effects of diC8‐PIP_2_ on TMEM16A and TMEM16B currents. We found that in the absence of intracellular Ca^2+^ (nominally Ca^2+^‐free intracellular solution), diC8‐PIP_2_ exhibited no effect on the TMEM16A and TMEM16B currents at all tested *V*_*m*_ (Figure 3A*ii*, B*ii* and Suppl. Figure S2 for expanded version of the image). This indicates that the application of diC8‐PIP_2_ cannot lead to activation of TMEM16A channel in the absence of intracellular Ca^2+^. Furthermore, the data demonstrate a lack of inhibition of the small endogenous currents in cells transfected with TMEM16B and in Ca^2+^ free solution.

### Sensitivity of TMEM16A and TMEM16B channels to endogenous PIP_2_

To test whether endogenous PIP_2_ modulates TMEM16A and TMEM16B currents, cells were co‐transfected with either TMEM16A or TMEM16B channels in conjunction with the membrane‐localized protein Danio rerio voltage‐sensitive phosphatase (DrVSP), which depletes endogenous PIP_2_ content by dephosphorylation when *V*_*m*_ is brought to depolarized values (Okamura *et al.,* 2009).

**Figure 4:**
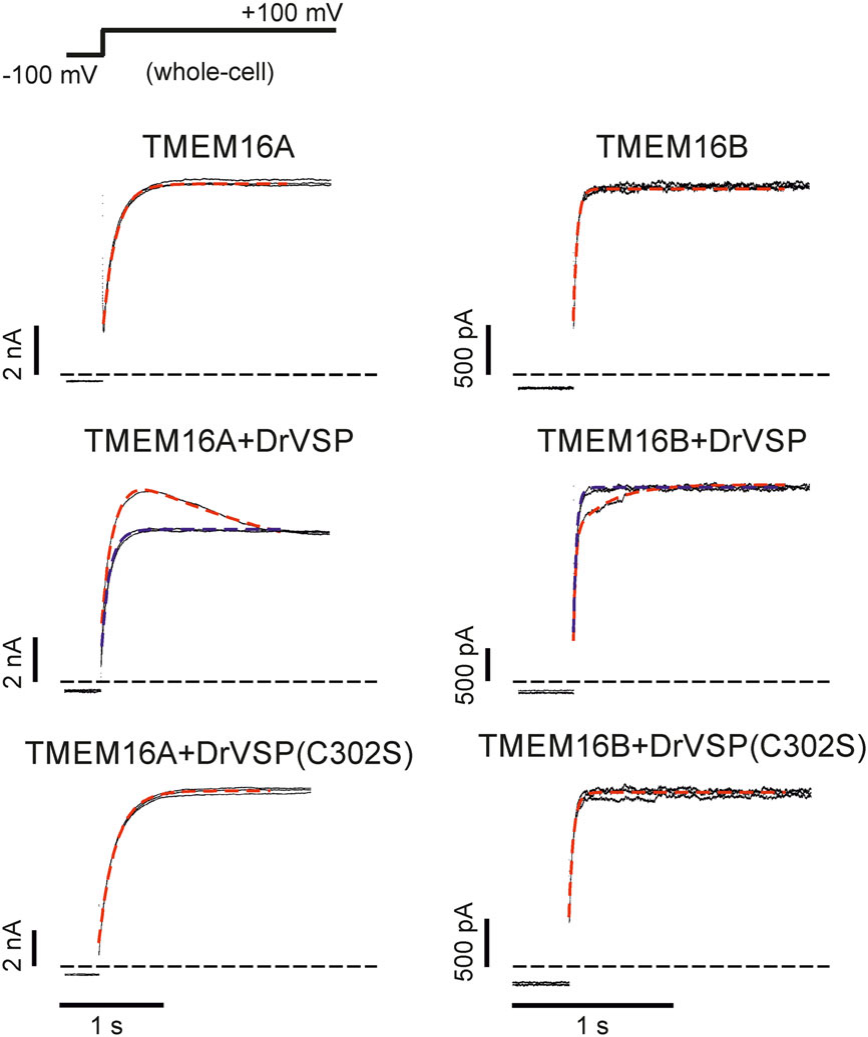
Effects of DrVSP activation on the kinetics of TMEM16A and TMEM16B currents. Whole‐cell currents recorded from HEK‐293T cells expressing either TMEM16A or TMEM16B alone, or co‐transfected with either DrVSP or DrVSP(C302S), as indicated. [Ca^2+^]_i_ was 0.3 or 0.6 μM for experiments involving TMEM16A or TMEM16B channels respectively. The stimulation protocol is shown in the top left corner. Each panel represents the current elicited by three consecutive voltage pulses from −100 to +100 mV (inter‐pulse duration was 0.5 s). Dashed Red traces represent the mono‐ or bi‐exponential fit of the currents elicited by the first pulse. Dashed blue traces represent the mono‐exponential fit of the currents elicited by the third pulse. Horizontal dashed black lines represent zero‐current levels.

Initial control experiments were carried out in the absence of DrVSP. Under this condition, when *V*_*m*_ was stepped to +100 mV for 4 s from the holding potential of −100 mV, large TMEM16A and TMEM16B whole‐cell currents were elicited that reached a stable steady‐state value (Figure 4). As previously reported, the TMEM16A currents activated more slowly than TMEM16B currents (Adomaviciene *et al.,* 2013; Scudieri *et al.,* 2013; Cruz‐Rangel *et al.,* 2015). The rate of TMEM16A and TMEM16B current activation was quantified by fitting the currents with a single exponential function with time constant τ_f_. The τ_f_ for TMEM16A currents was ~5 times greater than that for TMEM16B currents (Figure 4, Table 3). This stimulation pulse was repeated three times, with 0.5 s intervals between each stimulation. During these stimulations, the kinetics of the TMEM16A and TMEM16B currents remained unchanged (Figure 4 and Table 3). The amplitude of the steady‐state TMEM16A current was 473 ± 70 pA/pF (*n* = 8, first pulse); 473 ± 69 pA/pF (*n* = 8, second pulse) and 488 ± 69 pA/pF (*n* = 8, third pulse). The steady‐state TMEM16B current was 113 ± 15 pA/pF (*n* = 8, first pulse); 114 ± 15 pA/pF (*n* = 8, second pulse) and 115 ± 16 pA/pF (*n* = 8, third pulse).

**Table 3:** Parameters obtained from single or double exponential fit of the TMEM16A and TMEM16B currents elicited by three consecutive pulses to +100 mV

	Order of the pulse			
	First		Second	Third
	*τ*_*f*_ (ms)	τ_s_ (ms)	*τ*_*f*_ (ms)	*τ*_*f*_ (ms)
TMEM16A	143 ± 10 (*n* = 8)	−	151 ± 10 (*n* = 8)	158 ± 12 (*n* = 8)
TMEM16A + VSP	157 ± 19 (*n* = 14)	468 ± 84 (*n* = 14)	134 ± 13 (*n* = 14)	130 ± 15 (*n* = 14)
TMEM16A + VSP(C302S)	173 ± 22 (*n* = 5)	−	182 ± 28 (*n* = 5)	186 ± 28 (*n* = 5)
TMEM16B	27 ± 2 (*n* = 8)	−	26 ± 2 (*n* = 8)	25 ± 1 (*n* = 8)
TMEM16B + VSP	24 ± 2 (*n* = 12)	624 ± 23 (*n* = 12)	35 ± 2 (*n* = 12)	33 ± 2 (*n* = 12)
TMEM16B + VSP(C302S)	29 ± 2 (*n* = 8)	−	29 ± 2 (*n* = 8)	29 ± 2 (*n* = 8)

TMEM16 channels are either expressed on their own or in combination with DrVSP or DrVSP(C302S), as indicated

When cells were co‐transfected with TMEM16A and DrVSP, the first depolarizing step to +100 mV elicited a whole‐cell current with a biphasic component. In these experiments, the holding *V*_*m*_ was −100 mV to maintain DrVSP inactive. During the depolarizing step, the current reached a maximal point [187 ± 53 pA/pF (*n* = 14)] and then relaxed to a lower amplitude steady‐state level of 136 ± 39 pA/pF (*n* = 14) (Figure 4). This current was fitted with a double exponential function, with time constants τ_f_ of ~160 ms and τ_s_ of ~470 ms (Figure 4 and Table 3). The following two stimulations, however, gave rise to currents that were well described by a single exponential function with τ_f_ of ~130 ms (Table 3) and steady‐state values of 137 ± 40 pA/pF (*n* = 14) and 138 ± 38 pA/pF (*n* = 14) respectively. These values were indistinguishable from the current amplitude of ~136 pA/pF reached at the end of the first stimulus.

The same stimulation protocol was used to examine the current activation kinetics in HEK‐293T cells co‐transfected with TMEM16B and DrVSP. It was found that the first stimulation elicited a biphasic whole‐cell current that increased to a final, steady‐state value (Figure 4). This biphasic kinetics was characterized by τ_f_ of ~25 ms and τ_s_ of ~620 ms (Figure 4 and Table 3). The current amplitude at the initial transient plateau was 85 ± 12 pA/pF (*n* = 12), and steady‐state current at the end of the pulse was 94 ± 12 pA/pF (*n* = 12). In contrast, the subsequent two stimulations elicited currents with a single exponential time course with τ_f_ of ~30 ms in each case (Table 3). The steady‐state current amplitude was 96 ± 13 pA/pF (*n* = 12) and 97 ± 13 pA/pF (*n* = 12) for the second and the third pulse, respectively (Figure 4).

We interpreted the biphasic current time course of TMEM16A and TMEM16B currents observed in response to the first stimulation as being the result of the depletion of endogenous PIP_2_ by DrVSP. The subsequent stimulations would not manifest these effects, as endogenous PIP_2_ would already be depleted. We tested this idea by examining the consequence of co‐transfecting TMEM16A or TMEM16B channels with a mutant form of DrVSP, which does not support PIP_2_ dephosphorylation (Imai *et al.,* 2012). This DrVSP has the cysteine at position 302 mutated into serine and was termed DrVSP(C302S). When DrVSP(C302S) was present, each stimulation to +100 mV gave rise to TMEM16A and TMEM16B currents with single exponential kinetics indistinguishable from currents recorded in the absence of DrVSP. The TMEM16A steady‐state current amplitude was 355 ± 140 pA/pF (*n* = 5), 363 ± 144 pA/pF (*n* = 5) and 364 ± 142 pA/pF (*n* = 5) for the first, second and third pulse respectively (Figure 4, Table 3). The TMEM16B steady‐state current amplitude was 115 ± 17 pA/pF (*n* = 8), 118 ± 19 pA/pF (*n* = 8) and 122 ± 20 pA/pF (*n* = 8) for the first, second and third pulse respectively (Figure 4, Table 3).

### Combined effects of DrVSP and PIPK on TMEM16A and TMEM16B channels

**Figure 5:**
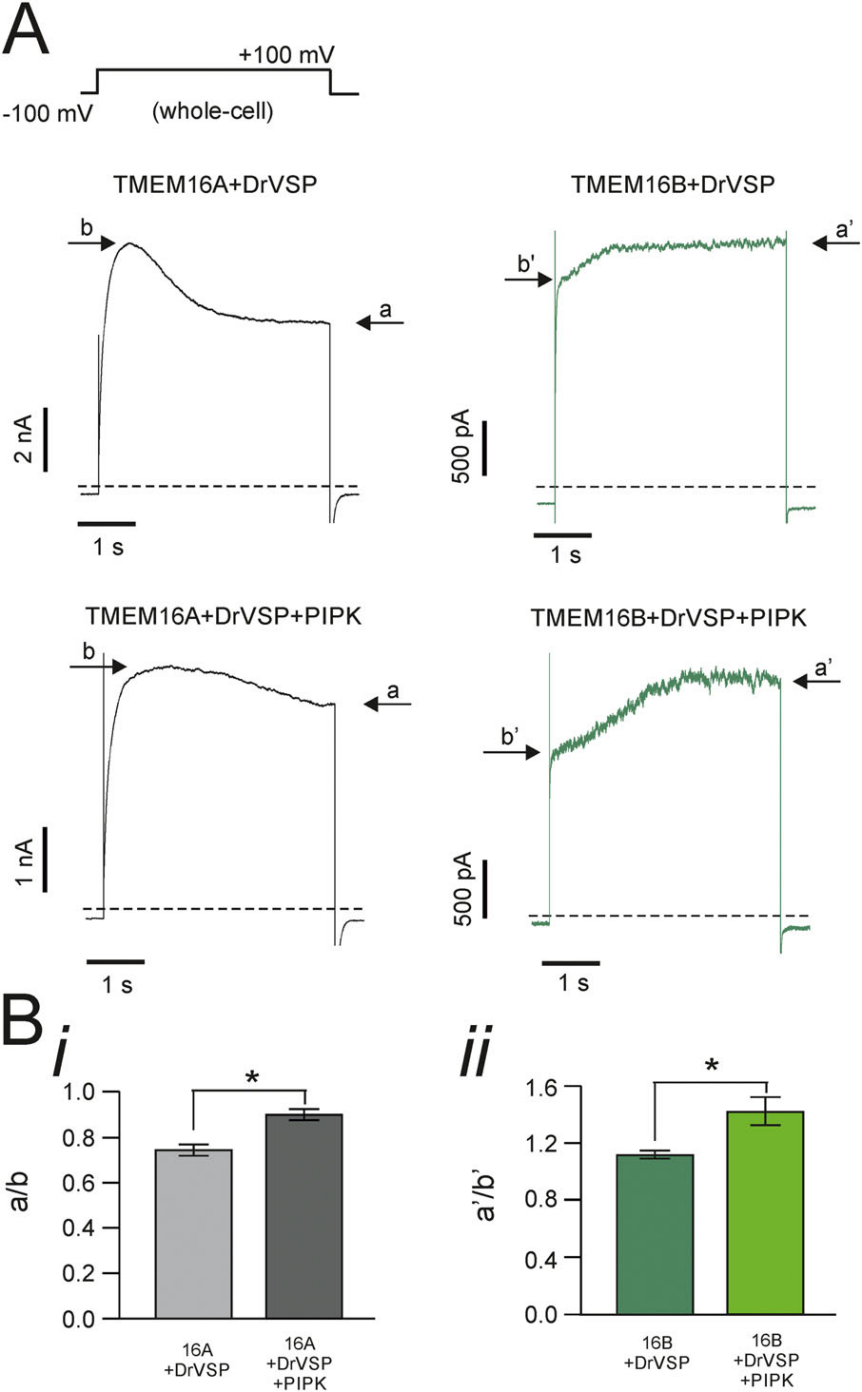
Combined effects of DrVSP and PIPK on TMEM16A and TMEM16B currents. (A) Whole‐cell currents obtained from HEK‐293T cells co‐transfected with TMEM16A or TMEM16B, DrVSP and PIPK, as indicated. [Ca^2+^]_i_ was 0.3 or 0.6 μM for experiments involving TMEM16A or TMEM16B channels respectively. The stimulation protocol is shown in the top left corner. Dashed lines represent zero‐current levels. Arrows indicate the steady‐state current (a) and the peak current (b) during the depolarizing pulse. (B, panel i) Mean extent of TMEM16A current inhibition measured as the ratio between current (a) and (b) in HEK‐293T cells co‐transfected with TMEM16A and DrVSP (*n* = 14) or with DrVSP and PIPK (*n* = 14); (B, panel ii) Mean extent of current activation measured as the ratio between current a' and b' in HEK‐293T cells co‐transfected with TMEM16B and DrVSP (*n* = 12) or TMEM16B with DrVSP and PIPK (*n* = 12). * *P* < 0.05 (paired *t*‐test).

To further test the hypothesis that the effects of DrVSP on TMEM16A and TMEM16B currents were due to endogenous PIP_2_ depletion, cells were co‐transfected with either TMEM16A or TMEM16B in conjunction with DrVSP and PIPK. This was done with the rationale that the presence of PIPK would oppose the effect of DrVSP by synthesizing additional PIP_2_. Whole‐cell currents were recorded in response to a single depolarizing step to +100 mV for 4 s from a holding potential of −100 mV (Figure 5A). These whole‐cell currents had kinetics involving multiple components and could not be satisfactorily described by a double exponential function; at least the sum of three exponentials was required to fit these currents (not shown). We measured the extent of DrVSP‐mediated inhibition of TMEM16A currents in the absence and presence of PIPK as the ratio between the peak current and the current measured at the end of the depolarizing pulse. This ratio was 0.75 ± 0.03 (*n* = 14) and 0.90 ± 0.02 (*n* = 14) in the absence and presence of PIPK respectively (Figure 5B). Thus, the presence of PIPK reduced the TMEM16A current inhibition provoked by DrVSP. We also examined the combined effect of DrVSP and PIPK on TMEM16B channels (Figure 5A). This was assessed as the ratio of the current measured at the beginning and at the end of the depolarizing pulse. This ratio was 1.12 ± 0.02 (*n* = 12) and 1.42 ± 0.10 (*n* = 12) in the absence and presence of PIPK respectively (Figure 5B). Thus, an elevated level of PIP_2_ caused by PIPK inhibited TMEM16B currents, and this allowed greater scope for activation when the cell was depleted of PIP_2_ by DrVSP.

### Recovery of the DrVSP‐mediated inhibition of TMEM16A and TMEM16B channels

**Figure 6:**
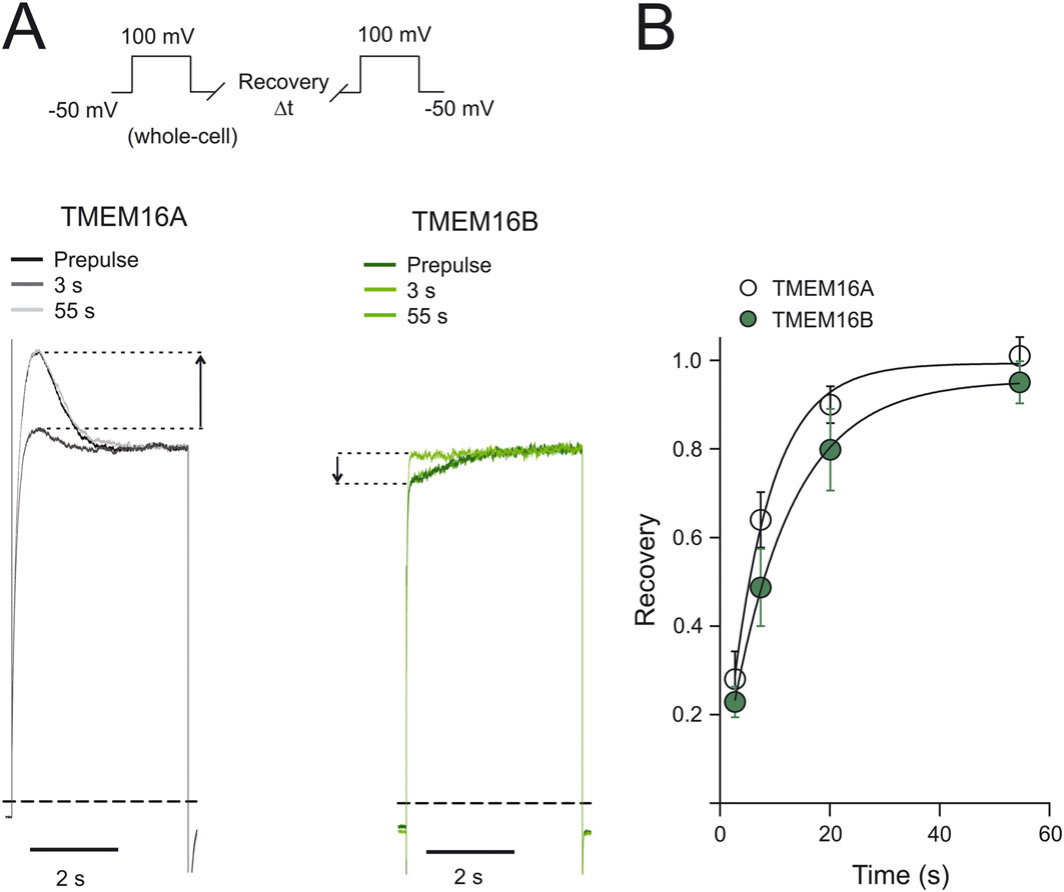
Time course of the recovery of TMEM16A and TMEM16B currents in response to DrVSP activation. (A) Whole‐cell currents recorded from HEK‐293T cells expressing TMEM16A or TMEM16B, and DrVSP, as indicated. Currents were elicited using the double‐pulse protocol described in Methods and illustrated in the top left corner. In each panel, the current elicited by a pre‐pulse was superimposed over the current elicited by two test pulses following recovery periods of different durations (3 and 55 s). To facilitate visual comparison, currents were normalized for the steady‐state current reached during each pulse. Dashed lines represent zero‐current levels. (B) Mean relationship between the extent of recovery of the DrVSP‐mediated modulation of the currents and the recovery time. The number of experiments was 11 (for TMEM16A and TMEM16B). The smooth curves through the points represent the best fit of the data with single exponential functions.

We argued that if the effects of DrVSP on the TMEM16A and TMEM16B currents were due to *bona fide* depletion of PIP_2_ from the plasma membrane of transfected HEK‐293T cells, these effects could be replicated if enough time was allowed for PIP_2_ synthesis to occur in the cells. This possibility was tested using a double‐pulse protocol (see Methods) during whole‐cell recordings in cells transfected with TMEM16A or TMEM16B and DrVSP (Figure 6). In these experiments, 1 mM of MgATP was included in the intracellular solution to enable PIP_2_ synthesis by endogenous phosphatidylinositol phosphate kinases. Furthermore, the holding potential was maintained at −50 mV, which is close to the resting membrane potential in HEK‐293T cells, while also being sufficient to maintain DrVSP inactivated (Okamura *et al.,* 2009). Figure 6 shows that the DrVSP‐mediated modulation of TMEM16A and TMEM16B currents is completely restored after about ≥50 s ‘recovery’ period at −50 mV. The relationship between the extent of recovery of this effect versus the duration of the time interval spent at −50 mV was characterized by a τ_r_ of 7.2 ± 0.7 s (*n* = 11) and 10.9 ± 2.5 s (*n* = 10) (N.S., *t*‐test) for TMEM16A and TMEM16B respectively.

## Discussion

The key finding of this study is the observation that PIP_2_ provokes opposing effects on TMEM16A and TMEM16B channels, leading to channel activation and inhibition respectively. These modulatory effects of PIP_2_ occurred in a concentration range similar to that causing modulation of other ion channel types including, but not limited to, KCNQ (e.g. Zhang *et al.,* 2003; Li *et al.,* 2011), K_v_ (e.g. Rodriguez‐Menchaca *et al.,* 2012) and TRPC1 (e.g. Saleh *et al.,* 2009) channels. The extent of PIP_2_ modulation of the TMEM16A current varied significantly depending on [Ca^2+^]_i_; in contrast, the extent of modulation of TMEM16B current did not vary over a broad range of [Ca^2+^]_i_ tested (0.6–2 μM). Furthermore, the effects of PIP_2_ on the TMEM16A current were observable at all *V*_*m*_ tested, while TMEM16B was only modulated at highly depolarized *V*_*m*_ > 50 mV. This suggests that *in vivo* modulation of TMEM16A currents by PIP_2_ may occur under resting conditions as well as at depolarized *V*_*m*_. In contrast, the effect of PIP_2_ on TMEM16B may only become relevant in the rare types of excitable cells that reach highly depolarized *V*_*m*_ > 50 mV during action potentials.

### Modulation of TMEM16A and TMEM16B channels by diC8‐PIP_2_

A recent study indicated that PIP_2_ binds directly to TMEM16A channels (Pritchard *et al.,* 2014). This study also included functional evidence that PIP_2_ inhibited native CaCC currents in isolated rPASMCs. In this published study, however, the functional effects of PIP_2_ on cloned TMEM16A channels were not investigated. Thus, we set out to study the extent of modulation of the heterologously expressed TMEM16A channel (and the closely related TMEM16B channel) by diC8‐PIP_2_ as well as endogenous PIP_2_. The use of heterologous expression systems allows the effects of channel modulation to be examined in the absence of potential additional tissue‐specific modulatory pathways. diC8‐PIP_2_ enhanced cloned TMEM16A currents recorded from excised inside‐out patches and depletion of endogenous cellular PIP_2_ inhibited whole‐cell currents. It is known that TMEM16A is an essential component of CaCC in rPASMCs (Manoury *et al.,* 2010). However, the possibility that in rPASMCs the TMEM16A channels are associated with endogenous binding partners or combine with other TMEM16 members to form channels with novel regulatory properties cannot be excluded. Thus, cell specific components may be responsible for the differential regulation of TMEM16A channel in PASMCs and heterologous expression systems. At present, it is not known whether TMEM16A in other native cell types, including vascular smooth muscle cells from other circulations, is differentially modulated by PIP_2_. Defining this could be of considerable importance in understanding how potential pharmacological agents acting on PIP_2_ synthesis/depletion may affect TMEM16A in different cell types.

A previous study indicated that heterologous whole‐cell TMEM16A currents were insensitive to compounds that interfere with inositolphosphates and phosphatidylinositols (Tian *et al.,* 2013). Our observation that the effects of diC8‐PIP_2_ on TMEM16A currents were negligible at relative high (>2 μM) [Ca^2+^]_i_ could explain the lack of effect observed by Tian *et al.* (2013). In this study, whole‐cell TMEM16A currents were elicited in response to factors that result in high [Ca^2+^]_i_ such as ionomycin (Morgan and Jacob, 1994) or prolonged exposure of cells to extracellular ATP (Qi *et al.,* 2000).

In our study, we also observed that TMEM16B currents were inhibited by diC8‐PIP_2_ in the same concentration range that activated TMEM16A. Both changes in TMEM16A and TMEM16B current amplitudes were due to changes in channel gating, while single channel conductance and ion selectivity remained unaltered. Thus, the diC8‐PIP_2_ acts as a gating modifier of cloned TMEM16A and TMEM16B channels.

The concentration range of diC8‐PIP_2_ that modulated the TMEM16A and TMEM16B currents [1–100 μg·mL^−1^ (1.17–117 μM)] is similar to the reported concentration of endogenous PIP_2_. Reported values of membrane PIP_2_ concentration include ~2–30 μM in cultured cell lines (McLaughlin *et al.,* 2002), ~50 μM in unstimulated neutrophils (Stephens *et al.,* 1991) or 200 μM in platelets in the resting state (Hartwig *et al.,* 1995).

### Modulation of TMEM16A and TMEM16B channels by endogenous PIP_2_

The voltage‐sensitive protein phosphatase DrVSP was used to assess the sensitivity of the TMEM16A channel to endogenous PIP_2_. DrVSP is a membrane‐resident phosphoinositide 5‐phosphatase that enables rapid depletion of PIP_2_ content in intact cells when *V*_*m*_ is brought from negative to positive values (Okamura *et al.,* 2009). Specifically, and consistently with the modulatory effects of diC8‐PIP_2_ in inside‐out patches, DrVSP activation reduced the amplitude of TMEM16A currents, whereas TMEM16B current amplitude was increased.

The kinetics of PIP_2_ depletion by DrVSP have been investigated using fluorescence resonance energy transfer imaging of PIP_2_ levels in HEK‐293T cells (Itsuki *et al.,* 2014). It was shown that within ~1 s of activation of the phosphatase, PIP_2_ in the membrane was significantly depleted (Itsuki *et al.,* 2014). This time course of alterations in plasmalemmal PIP_2_ content is consistent with the changes in current amplitude we observed in cells expressing TMEM16A or TMEM16B channels and DrVSP. The effects of DrVSP on TMEM16A and TMEM16B currents were abolished when a second depolarizing pulse was elicited after ~0.5 s. We interpret this loss of modulation as due to the fact that PIP_2_ has been depleted during the first pulse. Consistent with this idea was the fact that the DrVSP‐dependent modulations of TMEM16A and TMEM16B currents were fully re‐established after ~55 s at −50 mV. This duration corresponds to the estimated time of PIP_2_ re‐synthesis by endogenous PIPK (Loew, 2007; Falkenburger *et al.,* 2010; Itsuki *et al.,* 2014).

The reduction in the effects of DrVSP on TMEM16A currents caused by overexpression of PIPK is qualitatively consistent with an increased amount of PIP_2_ being present in the cell. On the other hand, the increased basal amount of PIP_2_ might have rendered the effects of DrVSP on TMEM16B more pronounced: a higher initial extent of current inhibition caused by increased basal levels of PIP_2_ would provide greater scope for current activation following DrVSP activation.

### Towards the identification of PIP_2_ binding site(s) in TMEM16A and TMEM16B channels

The TMEM16A or TMEM16B current versus [diC8‐PIP_2_] relationships we have determined do not provide a direct indication of diC8‐PIP_2_ affinity. The mid‐points of these curves are presumably influenced by competition between diC8‐PIP_2_ and endogenous PIP_2_, the exact concentration of which was unknown. The parameters *j* and *h* of the Hill fit of these relationships equalled ~1 in each case, which may be suggestive of a similar number of diC8‐PIP_2_ molecules binding to the TMEM16A and TMEM16B channels.

In general, PIP_2_ modulates ion channels by binding to a diverse range of recognition domains, albeit with different specificities and potencies (Lemmon, 2003; Gamper and Shapiro, 2007; Huang, 2007; Hansen, 2015; Hille *et al.,* 2015). Recognition domains include pleckstrin homology domains, myristoylated alanine‐rich C‐kinase substrate domains, phox homology domains, FYVE zinc finger domains, epsin N‐terminal homology domains and 4.1 protein‐ezrin‐radixin‐moesin domains. These domains differ significantly in structural conformation, size and specificity (Lemmon, 2003; Gamper and Shapiro, 2007; Huang, 2007; Hansen, 2015; Hille *et al.,* 2015). The precise set of residues involved in PIP_2_ binding cannot be directly identified through analysis of the TMEM16A and TMEM16B primary structure. This is because typically PIP_2_ binding sites involve residues that are distant in protein primary structures but may be positioned next to each other in their tertiary structures. Understanding the structural determinants of TMEM16A and TMEM16B channels involved in PIP_2_ binding will be an important pursuit for future research.

### Pharmacological and pathophysiological significance

The extent to which PIP_2_ modulation of TMEM16A or TMEM16B channels affects the cell electrical activity may vary depending on the cell type. Factors that may determine the impact of this modulation on cell electrical activity may include (1) the proximity of TMEM16A or TMEM16B channels to cellular mechanisms that determine membrane PIP_2_ contents (such as PLC or PIPK), the abundance and distribution of which may vary from cell type to cell type and (2) the contribution that TMEM16A or TMEM16B channels play to the electrical activity of an individual cell type, which may depend on factors such as channel expression and the complement of other transport mechanisms being present.

PIP_2_ levels are dynamically regulated in living cells, depending on the extent of PIP_2_‐depleting and PIP_2_‐synthesizing mechanisms. For example, muscarinic stimulation of sympathetic neurons leads to significant dynamic variations in PIP_2_ levels (Kruse *et al.,* 2016). It is noteworthy that in some cell types, such as mouse portal vein smooth muscle cells, TMEM16A appear to localize in caveolin‐1 containing plasma membrane lipid rafts (Sones *et al.,* 2010). These are regions of the membrane that also tend to concentrate a variety of receptors, including GPCRs (Insel and Patel, 2009). Furthermore, in cell types such as nociceptive sensory neurons, TMEM16A localized to the same membrane fraction as GPCRs such as the bradykinin B_2_ receptor, protease‐activated receptor PAR2 and also with caveolin‐1, a lipid raft marker (Jin *et al.,* 2013). Thus, in these cell types, TMEM16A may be surrounded by a local membrane environment in which changes in PIP_2_ concentration may vary dynamically in the immediate vicinity of the TMEM16A channel.

Interfering pharmacologically with PIP_2_ signalling could lead to modulation of TMEM16A or TMEM16B channel activity. Pharmacological modulators of TMEM16A and TMEM16B channels would constitute important tools for scientific research and potentially for therapeutic treatment of conditions associated with altered Cl^−^ transport. For instance, TMEM16A channels have been proposed as possible therapeutic targets for respiratory diseases of impaired mucus clearance, including cystic fibrosis, chronic obstructive pulmonary disease and asthma (Huang *et al.,* 2012b; Sondo *et al.,* 2014; Sala‐Rabanal *et al.,* 2015). The importance of TMEM16A in epithelial cell function is emphasized by the observation that mice in which the TMEM16A gene has been deleted show a strongly reduced Ca^2+^‐dependent Cl^−^ secretion, accumulation of mucus in the airways and impaired mucociliary transport (Ousingsawat *et al.,* 2009; Rock *et al.,* 2009). Furthermore, Th‐2 cytokines‐driven goblet cell hyperplasia, a feature of asthma and other respiratory diseases, leads to alteration of TMEM16A expression in human cells and consequent alteration in bicarbonate transport; this in turn affects mucus properties (Gorrieri *et al.,* 2016). TMEM16A channels are also abundantly expressed in arterial smooth muscle. Overexpression of TMEM16A has been reported in pulmonary arteries during pulmonary hypertension (Sun *et al.,* 2012), and up‐regulation of Cl^−^ currents has been implicated in the proliferation of PASMCs (Liang *et al.,* 2009). Thus, agents that reduce TMEM16A activity could be beneficial in treating pulmonary hypertension by inducing smooth muscle relaxation and possibly by reducing cell proliferation.

We have shown that PIP_2_ modulates TMEM16B channels, which are especially relevant in hippocampal neurons (Huang *et al.,* 2012c), olfactory neurons and photoreceptors (Stephan *et al.,* 2009; Stohr *et al.,* 2009; Hengl *et al.,* 2010; Pietra *et al.,* 2016). In these cell types, however, the peak of the action potential does not overshoot the 50 mV. Thus, PIP_2_ modulation in these cells is unlikely to occur under physiological conditions. TMEM16B is also expressed in DRG neurons (Zhao *et al.,* 2016), which reportedly are characterized by a peak of AP of ~55 mV, and this value may be slightly elevated in the presence of gain‐of‐function mutations in voltage‐gated sodium (Na_v_) channels (Dib‐Hajj *et al.,* 2008; Hoeijmakers *et al.,* 2012). Elevation of the peak of action potential might also occur during hypernatraemia, which shifts the Na^+^ equilibrium potential towards higher values. It is conceivable that gain‐of‐function mutations in voltage‐gated calcium (Ca_v_) channels might also be associated with an increased action potential peak. Thus, potential PIP_2_‐mimicking drugs might interfere with TMEM16B channels under these types of pathological conditions and might be an important consideration in terms of safety pharmacology under these special circumstances.

## Author contributions

P.T. conceived and designed the study. All authors designed and performed individual experiments and analysed the data. P.T. and C.M.T drafted the manuscript, while all authors reviewed and approved the final version of the manuscript.

## Conflict of interest

The authors declare no conflicts of interest.

## Declaration of transparency and scientific rigour

This Declaration acknowledges that this paper adheres to the principles for transparent reporting and scientific rigour of preclinical research recommended by funding agencies, publishers and other organisations engaged with supporting research.

## Supporting information


**Figure S1** Effects of diC8‐PIP_2_ on TMEM16A and TMEM16B currents elicited by 78 μM [Ca^2+^]_i_. A. Currents recorded from inside‐out patches excised from HEK‐293T cells expressing either TMEM16A or TMEM16B, as indicated. diC8PIP_2_ [100 μg·mL^−1^ (117 μM)] was applied as indicated by the horizontal bars. The *V*_*m*_ was keep at +70 mV for the entire duration of the recordings. The dashed lines represent the zero‐current level. B. Mean TMEM16A or TMEM16B currents measured in the presence of diC8‐PIP_2_ normalized to the currents measured in the absence of diC8‐PIP_2_. The number of experiments was 8–12 in each case.
**Figure S2** Effects of diC8‐PIP_2_ on TMEM16A and TMEM16B currents in nominally Ca^2+^‐free intracellular solution. Mean TMEM16A or TMEM16B steady‐state current *versus V*_*m*_ relationships measured in the absence (control) or presence of diC8‐PIP2 [100 μg·mL^−1^ (117 μM)]. [Ca^2+^]_i_, was 0.
