# Supplementary material for: Contrasting effects of phosphatidylinositol 4,5‐bisphosphate on cloned TMEM16A and TMEM16B channels
Source: Br J Pharmacol. 2017 Aug 10;174(18):2984–99. doi: 10.1111/bph.13913 (PMC5573538; doi:10.1111/bph.13913)
Supplement: Supplementary file 1 — Figure S1 Effects of diC8‐PIP2 on TMEM16A and TMEM16B currents elicited by 78 μM [Ca2+]i. A. Currents recorded from inside‐out patches excised from HEK‐293T cells expressing either TMEM16A or TMEM16B, as indicated. diC8PIP2 [100 μg·mL−1 (117 μM)] was applied as indicated by the horizontal bars. The V m was keep at +70 mV for the entire duration of the recordings. The dashed lines represent the zero‐current level. B. Mean TMEM16A or TMEM16B currents measured in the presence of diC8‐PIP2 normalized to the currents measured in the absence of diC8‐PIP2. The number of experiments was 8–12 in each case. Figure S2 Effects of diC8‐PIP2 on TMEM16A and TMEM16B currents in nominally Ca2+‐free intracellular solution. Mean TMEM16A or TMEM16B steady‐state current versus V m relationships measured in the absence (control) or presence of diC8‐PIP2 [100 μg·mL−1 (117 μM)]. [Ca2+]i, was 0. [file BPH-174-2984-s001.pdf]

SUPPLEMENTARY INFORMATION

ADDITIONS TO THE METHODS

Electrophysiology

TMEM16A and TMEM16B currents were measured with the whole-cell or inside-out configuration of the patch-clamp technique using an Axon 200B amplifier (Molecular Devices, USA) controlled with GE-pulse software (http://users.ge.ibf.cnr.it/pusch/programs-mik.htm). Currents were filtered at 2-5 kHz and sampled at 10 kHz. Pipettes were prepared from borosilicate glass capillary tubes (Harvard Apparatus, USA) using a Narishige PC-10 pipette puller (Narishige, Japan). Pipette tip diameter yielded a resistance of ~2-3 MΩ in the working solutions. The bath was grounded through a 3 M KCl agar bridge connected to a Ag-AgCl reference electrode. In whole-cell recordings, the series resistance was usually compensated by ≥70 % to achieve a maximal effective series resistance generally lower than ~5 MΩ. To allow for equilibration of the pipette solution with the cell interior, whole-cell recordings started 5 min after establishing the whole-cell configuration (Manoury, Tamuleviciute & Tammaro, 2010). Experiments were conducted at 20-22 °C. The cell capacitance was assessed by measuring the area beneath a capacitive transient elicited by a 10 mV step or via the cell capacity compensation circuit of the amplifier. Current density was obtained by dividing the current amplitude for the cell capacitance. The exchange of solutions was achieved by using a local perfusion system consisting of 11 tubes of 1.2 mm diameter in which the tip of the patch pipette was inserted.

SUPPLEMENTARY FIGURES

Suppl. Fig. 1

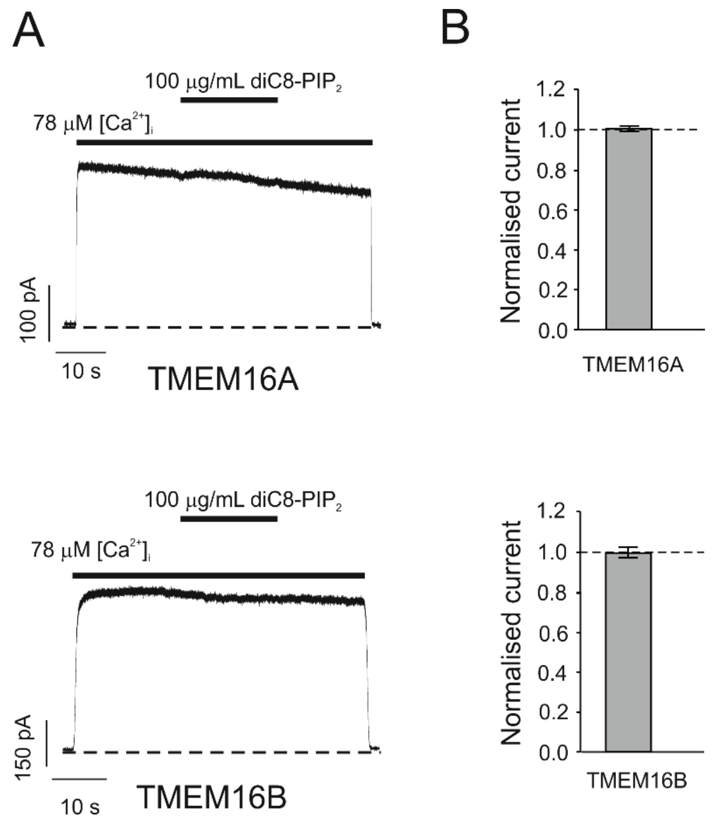

Suppl. Fig 1 Effects of diC8-PIP_2 on TMEM16A and TMEM16B currents elicited by 78 µM $[Ca^{2+}]_i$. **A.** Currents recorded from inside-out patches excised from HEK-293T cells expressing either TMEM16A or TMEM16B, as indicated. diC8-PIP_2 (100 µg/mL (117 µM)) was applied as indicated by the horizontal bars. The V_m was keep at +70 mV for the entire duration of the recordings. The dashed lines represent the zero-current level. **B.** Mean TMEM16A or TMEM16B currents measured in the presence of diC8-PIP_2 normalised to the currents measured in the absence of diC8-PIP_2. The number of experiments was 8-12 in each case.

Suppl. Fig. 2

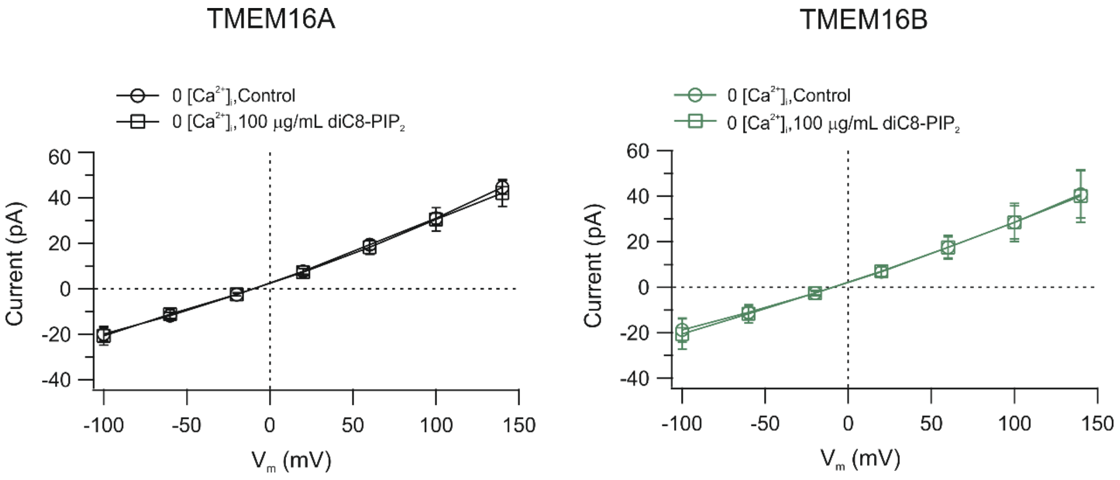

Suppl. Fig 2 Effects of diC8-PIP_2 on TMEM16A and TMEM16B currents in nominally Ca^{2+}-free intracellular solution. Mean TMEM16A or TMEM16B steady-state current *versus* V_m relationships measured in the absence (control) or presence of diC8-PIP_2 (100 µg/mL (117 µM)). $[Ca^{2+}]_i$, was 0.

REFERENCE LIST

Manoury B, Tamuleviciute A, & Tammaro P (2010). TMEM16A/Anoctamin 1 protein mediates calcium-activated chloride currents in pulmonary arterial smooth muscle cells. J Physiol 588**:** 2305-2314.
